# *Streptococcus pneumoniae* as a Cause of Salpingitis

**DOI:** 10.1155/S106474499400027X

**Published:** 1994

**Authors:** David Patterson, Celeste M. Johnson, Gilles R. G. Monif

**Affiliations:** Division of Laboratory Medicine and Infectious Diseases Department of Obstetrics and Gynecology Creighton University School of Medicine 601 North 30th Street, Suite 4810 Omaha NE 68131 USA

## Abstract

*Background:* A case of pneumococcal septicemia associated with laparoscopically documented acute salpingitis is reported.

*Case:* Gram-stained cul-de-sac pus revealed gram-positive encapsulated diplococci.

*Conclusion:* This case coupled with reanalysis of prior genital tract involvement in nonpregnant individuals argues that *Streptococcus pneumoniae* can mimic gonococcal diseases.


Infectious Diseases in Obstetrics and Gynecology 1:290–292 (1994)
© 1994 Wiley-Liss, Inc.

### *Streptococcus pneumoniae* as a Cause of Salpingitis

**David Patterson, Celeste M. Johnson, and Gilles R.G. Monif**

*Division of Laboratory Medicine and Infectious Diseases, Department of Obstetrics and Gynecology, Creighton University School of Medicine, Omaha, NE*

ABSTRACT

***Background:*** **A case of pneumococcal septicemia associated with laparoscopically documented acute salpingitis is reported.**

***Case:*** **Gram-stained cul-de-sac pus revealed gram-positive encapsulated diplococci.**

***Conclusion:*** **This case coupled with reanalysis of prior genital tract involvement in nonpregnant individuals argues that *Streptococcus pneumoniae* can mimic gonococcal diseases.**

© 1994 Wiley-Liss, Inc.

KEY WORDS

*Pneumococcal septicemia,* acute salpingitis, etiology

*Streptococcus pneumoniae* is not a normal constituent of vaginal bacterial flora[1,2]; however, given the opportunity, *S. pneumoniae* can be a significant pathogen for the female genital tract.[2–6]

Classically, involvement with the female genital tract has occurred in conjunction with primary pulmonary disease, engenderment of bacteriurinemia, and subsequent metastatic involvement.[7,8] More recently, there is the growing perception that *S. pneumoniae,* when present within the vaginal flora at the time of parturition or rupture of the fetal membranes, has the ability to ascend and infect the amnionic sac and secondarily the fetus.[2–6]

Genital tract disease due to *S. pneumoniae* has been reported in nonpregnant females. Isolated cases of spontaneous pneumococcal peritonitis have been described.[9,10] Hadfield et al.[11] reported a case of a 46-year-old woman with bilateral tubo-ovarian masses. Biopsy specimens from both tubes and from the wall of the abscesses demonstrated gram-positive, lancet-shaped diplococci which were documented to be *S. pneumoniae* by immunoperoxidase staining. Rahav et al.[12] reported a case of postmenopausal pneumococcal tubo-ovarian abscess from which *S. pneumoniae* was recovered.

The purpose of this paper is to report a case of pneumococcal salpingitis documented by laparoscopy and comment on its significance in terms of the pathogenetic spectrum attributable to *S. pneumoniae* as it involves the female genital tract.

### CASE REPORT

Initially, the patient (age 24) was transferred from jail to the emergency room because of severe abdominal cramping. The diagnosis of systemic lupus erythematosis had been previously documented, resulting in the patient's being placed on prednisone, 5 mg/day. The pelvic examination performed at that time was normal and she was diagnosed as having viral gastroenteritis. Two days later, because of a suicidal gesture involving excess self-administration of the antidepressant medication fluoxetine hydrochloride (Prozac), the patient was brought back to the hospital and admitted. At that time, she presented as a well-nourished, well-developed black female in no acute distress; however, vital signs included a temperature elevation of 39.8°C, pulse 116, respiration 16, and blood pressure 104/60. Pertinent physical findings included areas of spotty hyperpigmentation of the

Address correspondence/reprint requests to Dr. Gilles R.G. Monif, Department of Obstetrics and Gynecology, Creighton University School of Medicine, 601 North 30th Street, Suite 4810, Omaha, NE 68131.

Received December 8, 1993

Accepted March 28, 1994

forehead and malar area, forearms, hands, and feet consistent with discoid lupus. An abdominal examination was within normal limits. The pelvic examination was deferred. The chest X-ray, sinus films, and a computed tomography (CT) of the head were all normal. Laboratory evaluation revealed a white blood cell (WBC) count of 2,100 with a marked shift to the left, hemoglobin 9.8 g, hematocrit 29%, and platelets 90,000. Her electrolytes, liver function test, and arterial blood gases were within normal limits. Her drug screen was positive for acetaminophen. A fluorescent antinuclear antibody test (FNA) was positive with an antinuclear antibody (ANA) titer of 1/1,280 and an antinative DNA titer of 1/80. The $C_3$, $C_4$, and $C_5$ fractions of complement were low. The patient was placed under observation, but she continued to run a febrile course. Blood cultures, urine cultures, and throat cultures done at the time of admission were negative.

On the 3rd hospital day, the patient developed a sudden onset of severe cramping lower quadrant pain. An abdominal examination revealed diffuse direct and rebound tenderness. Cervical motion tenderness was demonstrated on pelvic examination. Cultures for *Neisseria gonorrhoeae*, *Chlamydia trachomatis*, and wet mount preparations for *Trichomonas vaginalis* were negative. Serological testing for human immunodeficiency virus (HIV)-1 and -2 was negative. The venereal disease research laboratory (VDRL) was nonreactive. Consultation was obtained from the Department of Obstetrics and Gynecology. At the time of this examination, the patient had a temperature elevation of 40.06°C, pulse 127, respiration 20, and blood pressure 118/50. Physical examination revealed four-quadrant tenderness to direct palpation and rebound tenderness. The patient refused a pelvic examination. All blood cultures drawn grew out *S. pneumoniae*. A pelvic ultrasound revealed a 5 × 5 × 2-cm complex mass with fluid collection in the cul-de-sac. Consent was obtained and laparoscopy performed under general anesthesia. Preoperatively, the patient was placed on ampicillin/sulbactam (Unasyn) 1.5 g q 6 h for 3 doses. At the time of surgery, the fallopian tubes were noted to be hyperemic and swollen. Purulent material could be expressed from both fallopian tubes. Approximately 300–400 cc of pus was present in the pelvis A gram-stained smear of this material revealed the presence of gram-positive, well-encapsulated, lancet-shaped diplococci which subsequently demonstrated a positive Quelling reaction. No organism was isolated; however, the culture techniques were inappropriate for the recovery of *S. pneumoniae*. The purulent material was drained from the cul-de-sac. An incidental appendectomy was performed. The pathology report revealed a normal appendix. Postoperatively, the patient continued to run a fever until 2 days after the removal of the Jackson-Pratt (JP) drains.

### DISCUSSION

Involvement of the female genital tract as a metastatic process secondary to maternal septicemia due to *S. pneumoniae* is a relatively well-documented phenomenon in the preantibiotic era.[1,7,8] The occurrence of cases of chorioamnionitis and/or perinatal septicemia in the absence of pulmonary involvement indicated the possibility of contiguous spread from the vaginal/cervical reservoir and subsequent involvement of the female upper genital tract.[3–6] The fallopian tubes in the 2 cases of spontaneous peritonitis due to *S. pneumoniae* in females were described as being swollen and hyperemic with pus emanating from the ends.[9,10] What was described is not a specific disease entity (spontaneous peritonitis) but more probably a progressive consequence of salpingitis.

Incrimination of *S. pneumoniae* as an etiological agent in cases of acute salpingitis is probably an underreported phenomenon. While *S. pneumoniae* will grow on 5–7% sheep blood agar culture when incubated in a $CO_2$ environment, the recovery of alpha hemolytic streptococci is usually not worked up any further and is often reported as "mixed vaginal flora." Recovery of alpha hemolytic streptococci from patients with acute salpingitis needs to be microbiologically evaluated to exclude the possibility that these isolates are *S. pneumoniae*.

The case in question coupled with those previously identified as being "spontaneous peritonitis" with isolation of *S. pneumoniae* from women with tubo-ovarian complexes indicates that *S. pneumoniae* can function as a primary pathogen for the fallopian tubes and that the entire pathogenic spectrum previously attributable to *N. gonorrhoeae* can also be mimicked by *S. pneumoniae*.

### REFERENCES

1. Alzahawi MF, Stack TA, Shrestha TL: Pneumococcal neonatal colonization and sepsis in association with maternal genital pneumococcal colonization. Br J Obstet Gynaecol 95:1198–1988.

2. Oxhorn H: The changing aspects of pneumonia complicating pregnancy. Am J Obstet Gynecol 70:1007–1010, 1955.
3. Westh H, Skibsted L, Korner B: *Streptococcus pneumoniae* infections of the female genital tract and in the newborn child. Rev Infect Dis 12:416–422, 1990.
4. Robinson EN Jr: Pneumococcal endometritis and neonatal sepsis. Rev Infect Dis 12:799–801, 1990.
5. Duff P, Gibbs RS: Acute intraamniotic infection due to *Streptococcus pneumoniae*. Obstet Gynecol 61:25–27, 1993.
6. DiNello CH, Chaisilwattana R, Fagnant RJ, Monif GRG: Neonatal *Streptococcus pneumoniae* septicemia and meningitis. J Reprod Med 35:297–298, 1990.
7. Nuckols HH, Hertig AT: Pneumococcus infection of the genital tract in women especially during pregnancy and the puerperium. Am J Obstet Gynecol 35:782–790, 1938.
8. Austrian R, Gold J: Pneumococcal bacteremia with special reference to bacteremia pneumococcal pneumonia. Ann Intern Med 60:759–760, 1964.
9. Jensen LS: Primary pneumococcal peritonitis. Ann Chir Gynaecol 73:95–96, 1984.
10. Gomez RJ, Padilla B, Delgado IA, Dargallo JL, Pedroviejo C, Elviro J: *Streptococcus pneumoniae* peritonitis secondary to genital tract infection in a previously healthy woman. Clin Infect Dis 15:1060–1061, 1992.
11. Hadfield TL, Neafie R, Lanoie LO: Tubo-ovarian abscess caused by *Streptococcus pneumoniae*. Hum Pathol 21: 1288–1289, 1990.
12. Rahav G, Ben DL, Persitz E: Postmenopausal pneumococcal tubo-ovarian abscess. Rev Infect Dis 13:896–897, 1991.
